# Intermittent auscultation versus continuous fetal monitoring: exploring factors that influence birthing unit nurses’ fetal surveillance practice using theoretical domains framework

**DOI:** 10.1186/s12884-017-1517-z

**Published:** 2017-09-25

**Authors:** Andrea M. Patey, Janet A. Curran, Ann E. Sprague, Jill J. Francis, S. Michelle Driedger, France Légaré, Louise Lemyre, Marie-Pascale A. Pomey, Jeremy M. Grimshaw, for the Canada Prime Plus team, Jeremy Grimshaw, Michelle Driedger, Martin Eccles, Jill Francis, Gaston Godin, Steve Hanna, Marie Johnston, France Légaré, Louise Lemyre, Marie-Pascale Pomey, Anne Sales

**Affiliations:** 1 Clinical Epidemiology Program, Ottawa Hospital Research Institute – General Campus, Ottawa, Canada; 2 School of Health Sciences, City, University of London, London, UK; 3 School of Nursing, Dalhousie University, Halifax, Canada; 4 Better Outcomes Registry and Network Ontario, Children’s Hospital of Eastern Ontario, Ottawa, Canada; 5 Department of Community Health Sciences, University of Manitoba, Winnipeg, Canada; 6 Département de Médecine Sociale et Préventive, Faculté de médecine, Université Laval, Québec, Canada; 7 Axe Santé des Populations et Pratiques Optimales en Santé, Centre de Recherche du CHU de Québec, Québec, Canada; 8 School of Psychology, University of Ottawa, Ottawa, Canada; 9 University of Montreal, Montreal, QC Canada; 10 Faculty of Medicine, University of Ottawa, Ottawa, Canada

**Keywords:** Labour, Fetal surveillance, Intermittent auscultation, Continuous fetal monitoring, Birthing Unit Nurses, Theoretical domains framework, Domains, Behaviour change

## Abstract

**Background:**

Intermittent Auscultation (IA) is the recommended method of fetal surveillance for healthy women in labour. However, the majority of women receive continuous electronic monitoring. We used the Theoretical Domains Framework (TDF) to explore the views of Birthing Unit nurses about using IA as their primary method of fetal surveillance for healthy women in labour.

**Methods:**

Using a semi-structured interview guide, we interviewed a convenience sample of birthing unit nurses throughout Ontario, Canada to elicit their views about fetal surveillance. Interviews were recorded and transcribed verbatim. Transcripts were content analysed using the TDF and themes were framed as belief statements. Domains potentially key to changing fetal surveillance behaviour and informing intervention design were identified by noting the frequencies of beliefs, content, and their reported influence on the use of IA.

**Results:**

We interviewed 12 birthing unit nurses. Seven of the 12 TDF domains were perceived to be key to changing birthing unit nurses’ behaviour The nurses reported that competing tasks, time constraints and the necessity to multitask often limit their ability to perform IA *(*domains *Beliefs about capabilities; Environmental context and resources)*. Some nurses noted the decision to use IA was something that they consciously thought about with every patient while others stated it their default decision as long as there were no risk factors (*Memory, attention and decision processes, Nature of behaviour)*. They identified positive consequences (e.g. avoid unnecessary interventions, mother-centered care) and negative consequences of using IA (e.g. legal concerns) and reported that the negative consequences can often outweigh positive consequences *(Beliefs about consequences).* Some reported that hospital policies and varying support from care teams inhibited their use of IA (*Social influences*), and that support from the entire team and hospital management would likely increase their use (*Social influences; Behavioural regulation*).

**Conclusion:**

We identified potential influences on birthing unit nurses’ use of IA as their primary method of fetal surveillance. These beliefs suggest potential targets for behaviour change interventions to promote IA use.

**Electronic supplementary material:**

The online version of this article (10.1186/s12884-017-1517-z) contains supplementary material, which is available to authorized users.

## Background

Fetal health surveillance is an obstetric intervention that has been in practice since the early 1800s [1]. Monitoring of fetal heart rate, a routine procedure typically carried out by Birthing Unit (BU) nurses [2], aims to assess fetal wellbeing and detect potential hypoxia during labour to prompt an intervention to reduce risk to both the fetus and mother [3]. Two types of fetal surveillance are typically employed throughout labour and birth. Intermittent auscultation (IA) is the practice of using an instrument to conduct the sound of the fetal heart through the maternal abdomen. Most commonly, some type of external hand-held or portable ultrasound transducer is used to listen immediately after a contraction for 1 min, every 15 to 30 min in active labour and every 5 min in the active portion of the second stage [4]. Electronic fetal monitoring (EFM) is the simultaneous use of an ultrasound transducer and a tocotransducer (to measure frequency and duration of contractions) continuously or for intermittent periods throughout labour [1]. The original purpose of EFM was to provide an early indication of a fetal stress or distress potentially requiring early obstetric intervention to prevent a compromised fetus. However, its use has contributed to an increase in maternal morbidities due to unnecessary medical interventions (e.g. caesarean section, instrumental vaginal births) without decreasing fetal/newborn morbidities [5].

While continuous EFM is easy to initiate and thought to provide clear readings from the printouts, the interpretation of information obtained from EFM is subject to disagreement between observers [6]. A systematic review of nine trials involving 18,561 women compared a policy of continuous EFM with IA and found that with continuous EFM there were higher rates of cesarean sections, operative vaginal deliveries [1] and lower women’s reported satisfaction with their birthing experience [2], but a lower frequency of neonatal seizures (although the seizures prevented by EFM were not associated with long term consequences [5]). EFM is also associated with about 70% of all legal claims concerning intrapartum care in relation to children with brain injury due to the variability in interpretation of recordings by medical professionals [2].

In addition to the published evidence supporting IA, Canadian and US obstetrics organizations, which publish guidelines for maternal and newborn healthcare, recommended IA as the method of fetal surveillance for all women [2, 4]. The Society of Obstetricians and Gynaecologists of Canada stated that there was insufficient evidence to justify the use of continuous EFM in routine practice, favouring IA as the preferred method of fetal surveillance for low risk women (i.e. women at term with a single live birth, with spontaneous onset of labour, no previous cesarean deliveries, no maternal medical, obstetrical, or intrapartum complications) [4]. The American College of Obstetrics and Gynaecology rescinded their original 1989 support of EFM and issued guidelines that promoted IA over continuous EFM for low risk women in labour [7]. Despite the scientific evidence, published guidelines, and support from obstetrics organizations supporting IA as the primary method of fetal surveillance, it is rarely used exclusively throughout labour in hospital births [7]. Continuous EFM is the most common obstetrics procedure in the United States [8] and 75% of women in Canada have continuous EFM while most of these women (70–80%) are low risk [9].

Many studies have explored both nurses’ and mothers’ beliefs underlying their fetal surveillance approach [3, 10–12]. However, few used a theoretical approach to inform their investigations. Application of theory in behaviour change research facilitates the identification of factors that may influence behaviour [13–17]. Theoretically-based interventions provide evidence about causal pathways of change, avoid implied causal assumption [18, 19] and have a better chance of yielding desirable changes [13, 14].

Attempts to predict, explain, and change behaviour have generated many theories and underlying constructs [15]. However, researchers have often tested a single or small number of theories, resulting in the exploration of only a small range of the psychological factors of behaviour [16, 17, 20–22]. Such studies can be uninformative if key factors are not represented in the theories that were applied. Hence, the Theoretical Domains Framework (TDF), which covers a comprehensive range of key psychological theories and constructs, has been used in a number of recent studies to identify key determinants of patient and professional behaviour and potential levers for change [23–29]. A group of behavioural scientists used a consensus approach to develop a framework from 33 theories of behaviour change (including motivation, action, and organizational theories; see Table 1 for details about the TDF) and 128 constructs [30]. The TDF (v1.0) includes 12 groups of key theoretical constructs, referred to as ‘theoretical domains’. We used the TDF to identify possible determinants (key domains) of BU nurses’ use of IA as their predominant method of fetal surveillance with healthy mothers during low risk labour.

**Table 1:** Domains from the TDF [30] and their descriptions adapted from Francis et al. [47]

Knowledge	Existing procedural knowledge, knowledge about guidelines, knowledge about evidence and how that influences what the participants do
Skills	Competence and ability about the procedural techniques required to perform the behaviour
Social/professional role and identity	Is the behaviour something the participant is supposed to do or someone else’s? (When discussing ‘we’/the collective) Boundaries between professional groups
Beliefs about capabilities	Perceptions about competence and confidence in doing the behaviour
Beliefs about consequences	Perceptions about outcomes and advantages and disadvantages of performing the behaviour or pervious experiences that have influenced whether the behaviour is performed or not
Motivation and goals	Priorities, importance, commitment to a certain course of actions or behaviours Intentions
Memory, attention and decision processes	Attention control, decision-making, memory, i.e. is the target behaviour problematic because people simply forget?
Environmental context/resources	How factors related to the setting in which the behaviour is performed (e.g. people, organisational, cultural, political, physical and financial factors) influence the behaviour
Social influences	External influence from other people, views of other professions, patients and families, doing what you are told and how that influences what you do
Emotion	How feelings, affect (positive or negative) may influence behaviour
Behavioural regulation	Ways of doing things that relate to pursuing and achieving desired goals, standards or targets
	Strategies the participants have in place to help them perform the behaviour
	Strategies the participants would like to have in place to help them
Nature of the behaviours	What is the participant’s history of the behaviour, have they any experience (done it often or not at all in the past), is the behaviour routine or automatic?

## Methods

### Design

This descriptive study used semi-structured interviews based on the TDF with nurses who work in BUs in Ontario to investigate their views about their fetal surveillance practices.

### Participants

Birthing Unit nurses, directly involved in intrapartum care for women in labour and representative of the three levels of birthing unit care provided in hospitals throughout Ontario, were selected using a convenience sampling strategy. Births in hospitals more often involve nurses who specialize in maternal and fetal care as well as obstetricians. Birthing unit or maternal care nurses in North America and Midwives in Europe hold the same roles, responsibility and training within a hospital environment. ‘Level 1’ hospitals typically provide care for the lowest risk mothers and babies (≥36 weeks gestation) and are commonly located in small and rural centres (but can also be found in cities). ‘Level 2’ hospitals provide care for mothers and babies ≥32 weeks gestation and usually have some specialists in house as well as neonatal intensive care beds. ‘Level 3’ hospitals care for women and babies with the highest risk and offer subspecialist care. The higher level-of-care centres do also provide care for low risk mothers and babies. Participants were contacted through a national maternity care email listserv facilitated by our clinical context expert (AES). They were provided with information about the purpose of the study and interested individuals were asked to contact the coordinator (AMP). Those nurses who showed interest were contacted via email and invited for an interview at a convenient time.

### Target behaviour and interview guide

We described the behaviour of interest using Fishbein’s TACT principle, whereby behaviour comprises four elements: Target, Action, Context, and Time [31]. The behaviour of interest was “using IA (action) as the primary method of fetal surveillance for a healthy woman (target) with a low risk pregnancy (context) during labour (time)”. Healthy women with a low risk pregnancy were defined as ‘women at term with a single live birth in cephalic presentation, with spontaneous onset of labour, no previous cesarean deliveries, no maternal medical problems, and no obstetrical or intrapartum complications’ [4].

An interview guide was developed, in collaboration with clinical and behaviour change researchers with expert knowledge of the TDF (JJF, AMP, JMG, JAC) and a content expert in the field of labour and birth (AS), based on the TDF to elicit beliefs from all 12 domains about the behaviour. After pilot testing with two BU nurses, basic wording of two questions from the draft interview guide was modified to improve the flow of the questions (See Additional file 1 for Interview Topic Guide). The reworded interview guide was used for the remainder of the interviews. All interviews, including the pilot interviews, were included in the analysis.

### Data collection

All interviews (undertaken by AMP) were conducted by phone. The interviews were digitally recorded and lasted on average 30 min. The recordings were transcribed verbatim and anonymised. We continued to recruit and interview BU nurses and used the concept of data saturation to determine when we no longer needed to continue interviewing. In other words, we conducted interviews until no new information was being offered [32, 33] which occurred after 12 interviews.

### Data analysis

Similar to other TDF studies [16, 24, 27, 34], the analysis was conducted in two stages. First, two researchers (AMP, JAC) coded each participant utterance onto one or more of the theoretical domains. Two pilot interviews were used to formulate a coding strategy. The two researchers coded the first pilot interview in collaboration to develop the coding strategy and the second pilot interview was used to ensure the two coders were comfortable with the developed strategy. Subsequent coding of the remaining interviews was completed independently using NVivo [35]. Interrater reliability (Kappa; κ) was calculated in NVivo for each interview to assess whether the two researchers coded the same response into the same domain [36, 37]. Although initial interrater reliability was calculated, all disagreements between researchers were resolved through the consensus process. In some instances utterances were coded in a single domain, in other instance where utterances would not be fully represented by a single domains, the utterance was coded into multiple domains [24].

In the second stage of analysis, one researcher (AMP) generated statements that represented the specific beliefs from participants’ responses (statements that summarise key concepts within each domain [16]). Specific beliefs that centred on the same theme or were polar opposites of a theme were grouped together and belief statements were worded to convey a meaning that was common to these participants’ utterances. A second researcher (JAC) reviewed the generated statements and groupings by themes to ensure accurate representation of content.

Key domains relating to the nurses’ use of IA were identified through discussion between two researchers (AMP, JAC) and confirmed by a health psychologist (JJF). In this paper “key domains” refers to those domains, which provide sufficient evidence to target in an intervention to change the behaviour. Briefly, three factors were considered to identify domains as key: 1) reported strength of opinion that the beliefs influenced the behaviour, 2) presence of conflicting beliefs, and 3) frequency of the beliefs across interviews. All of these factors were considered concurrently in establishing domain importance. For example, if the belief that ‘the skills required to use IA are not different than any other technique to monitor a baby’ was consistently reported, we concluded that lack of skills was unlikely a determinant of poor IA use. In contrast, if the majority of respondents reported the belief that ‘not enough Dopplers discourages IA use’ then the *Environmental context and resources* domain would have been selected as a potential determinant of poor IA use. Similarly, *Beliefs about consequences* would be identified as a key domain if conflicting statements about potential consequences associated with the behaviour ranged from negative to positive.

### Ethics, consent and permission

Ethics approval was obtained from the Ottawa Health Network Research Ethics Board (Protocol No. 2008237-01H). Consent was obtained from participants who agreed to be interviewed for the inclusion of their responses in analysis and reporting.

## Results

### Participants

Twelve female BU nurses from community (*n* = 7) and academic (*n* = 5) hospitals throughout Ontario participated in the interviews. These nurses represented the three levels of care provided at Ontario Hospitals (Level 1, *n* = 3; Level 2, *n* = 6; Level 3, n = 3) and their experience as a BU nurse ranged in years from 2 to 25 (median = 19 years).

### Interrater reliability

A total of 430 utterances from 12 interviews were coded into 12 domains. Interrater reliability for each interview ranged from κ = .77 to κ = .89 (mean ± SD; .84 ± .04) and thus had either ‘substantial agreement’ or ‘excellent agreement’ [36, 38]. Based on the principles for achieving data saturation from Francis et al. [33], no new shared beliefs were elicited in interviews 10, 11, and 12; therefore data saturation was achieved after 12 interviews.

### Key themes identified in domains

Seven domains (of the 12) were identified as potentially key to the decision to use IA as the predominant method of fetal surveillance (*Nature of behaviour; Beliefs about capabilities; Beliefs about consequences; Memory, attention, decision processes; Environmental context and resources; Social influences; Behavioural regulation)* [Table 2].

**Table 2:** Summary of belief statements and sample responses from Birthing Unit Nurses grouped by theoretical domains identified as key to influencing fetal surveillance

Domains	Specific belief	Sample responses	Frequency out of 12
Nature of behaviour	IA is part of my fetal surveillance for low risk women	“If she has no risk factors for herself or the baby and there should be IA.” (N1)	11
		“For me it [IA] is… part of my fetal surveillance.” (N2)	
		“Absolutely…IA is part of my surveillance.” (N4, N12)	
	I have been using IA for so long it’s just what we use.	“I think it’s just a pattern of habit that somebody is in front of you and you know if it’s an urgent situation or fast moving or a slow moving one you are talking and you are doing it at the same time so I’m just reaching for that and holding it on the belly. Like I can do that instantly without belts or wires or anything else. So I think force of habit, believing that’s what we should do and that it is the recommendation.” (N5)	4
		“I think it’s experience. I think experience makes you more comfortable with IA.” (N3)	
		“That’s what we use and that’s what we have used for years and we’ve not had a problem with it.”(N4)	
	I’ll use IA if the woman is healthy and there are no complications	“I would say almost all of them unless they have an epidural like going to the hospital setting and have epidural analgesia then they need to be monitored continuously.” (N12)	3
		“If a women is healthy. First baby, second baby or third baby doesn’t matter. If she has had previous uneventful pregnancies before. If she’s deemed to be low risk I mean there’s no issues with Mom or baby that warrant continuous fetal heart monitoring then I do do IA.” (N3)	
	There are complications with mother when IA is just not appropriate.	“So if we know what our decelerations are and everything through the fundamentals of fetal health self-learning package so we watch and listen for those okay with IA we just mostly listen. If we suspect that we’re getting decels when we shouldn’t which is post contraction then we hook them up to EFM to monitor if it’s a force of not just one, it’s just a ‘red flag’ to increase your IA surveillance." (N4)	3
		“I would say almost all of them unless they have an epidural like going to the hospital setting and have epidural analgesia then they need to be monitored continuously.” (N12)	
		“Well Oxytocin is ordered to induce or augment labour and we do quite a bit of that and so then it is policy that they are on continuous monitoring if they are on Oxytocin.” (N8)	
	Some people are set in their way with respect to fetal monitoring and will always do it the way they’ve done it.	“That always fascinates me and so I think more than busyness is that people’s entrenched like the way they’ve always done it before they just think no I’ve always done it this way and I’m going to keep doing it this way and it’s the more protective conservative way to do it every half hour versus every hour, but that more conservatism we know leads to greater interventions and more surgical births and all that so it’s interesting.” (N5)	2
		“There is some old school that have had difficulty giving up, and I’m old school, that initial observation strip on admission. There are some people who still do that.” (N9)	
	During the second stage of Labour it becomes more difficult to use IA	“The challenge becomes in the second stage and pushing for anything whether it’s IA or ESM.”(N1)	2
		“The second stage of labour we usually would have them connected because you need to see every second what’s going on.” (N2)	
Beliefs about Capabilities	I am comfortable using IA in healthy labouring women.	“So for me it’s more experience on both neonatal as well as the mom. I’m comfortable using it.” (N7)	10
		“I’m also comfortable doing it in that I feel like I am quite competent…” (N8)	
		“Yes, absolutely. I’m comfortable…[using IA in this patient population]” (N9, N4)	
	I am confident using IA	“…so you know, I have many years of that so I can almost anticipate what the outcome is going to be. If the outcome of the infant is not where we want it to be I’m confident that I can deal with that so that’s part of my education here as well.” (N7)	11
		“Well I’ve had 25 years experience so I’m comfortable and…I’m comfortable because I stay current in my practice and all the nurses do here." (N4)	
		“I feel reasonably confident, yes…I guess because I’ve been doing obstetrics for so long so you have some comfort level to know the rhythms and if I’m concerned about the fetal heart then you know that you can always put the mom on the continuous monitor right.” (N10)	
	It’s very easy/difficult to decide to use IA as my predominant method of surveillance.	“Well I think if everything goes well it’s easy to use.” (N10)	11
		“Personally I find it [IA] quite easy.” (N11)	
		“For me personally it’s not a problem…[to use IA as the predominant method of fetal surveillance with a healthy woman having a low risk pregnancy].” (N3)	
		“It is very difficult.” (N12)	
	It’s very easy because I have the right knowledge base, my experience, and the support of my team.	“As I said before having the waterproof monitors, having support from your obstetricians that they are coming around to IA being a very adequate source of monitoring.” (N8)	4
		“For me specifically it’s probably my knowledge base and my experience and I know I have the support of the physicians.” (N7)	
	It’s very hard to take a woman off a monitor and do IA once she’s been put on.	“I find it’s very hard to take a woman off the monitor once put on even if everything is okay.” (N11)	3
Beliefs about Consequences	Using IA is more time consuming and difficult to multitask	“Only that it’s more of a time constraint I think…[drawback to using IA].” (N8)	4
		“The drawbacks I think I know sometimes again depending on the acuity of the floor I find that it’s more time consuming than EFM because EFM is easier, it’s on you look, you document, you walk away. I think for the first line nurses it can be more time consuming and it’s unfortunate but you have to…” (N3)	
	Using IA increases the legal concern that you can’t provide a strip printout should something go wrong.	“I guess there’s always the legal concern about you can’t prove it so there’s a thought about well if you can show a good strip then that’s very supportive of your assessment of this patient. I think that’s probably the only thing.” (N1)	5
		"If something happened you don’t have that strip there that you can go back to and look at. It’s somewhat more time consuming for that reason." (N8)	
		“Well I am thinking immediately of a negative. If at the delivery there is an unexpected—a baby that has a longer than usual transition to—if the outgo basically is lower than one would expect there would be questions about whether the monitoring if it was just down to the experience and the reliability of the nurse to interpret reassuring the mum, reassuring her rate patterns with IA if the outgo was low then there would immediately be ‘oh heck were you really interpreting reliably?’ The question would immediately be asked and there would be no proof other than what the nurse was recording.” (N12)	
	Using IA provides the mother with support and a more positive experience	“Oh I think we would have more active women and more positive labours. It possibly has less observation of the woman.” (N8)	5
		“Positive wise I think you know again that supportive care, not being hooked up to a monitor, feeling confident in the nurse, you know that whole positive experience." (N7)	
		“It’s easy because the patients have better satisfaction, they are more mobile, they can get up to the shower, they can you know use alternate methods of pain relief, they are not strapped to a monitor and stuck in a bed.” (N6)	
	Using IA reduced methods of pain relief or interventions.	“Well I use it predominantly and I think that it increases patient satisfaction and decreases unnecessary intervention.” (N9)	4
		“… I feel anecdotally it reduces the number of requests for epidural and analgesia actually when the woman is up and moving about.” (N12)	
		“It’s a busy night and you are over monitoring and then you are lining up another section and the obstetrician is already busy and people are tired like it’s a win-win situation if we’re keeping things at an appropriate low level of intervention. Intervene when we need to but not when we don’t.” (N5)	
	Using IA encourages a more active labour where the mother is able to move and walk.	“I think the majority of the women that I have done IA on they much appreciate the fact that they are able to walk around." (N3)	4
		“…continuous EFM it does commit women to be relatively still and like in the same spot and position, well we can encourage them to change position but they are committed to the bed essentially or as long as the monitor as far as the monitor can tether them.“ (N2)	
	Using IA allows us (the nurses) to be more focussed on the mother and baby than EFM.	“It encourages rapport and to work with the woman. It requires a contact between the nurse and the woman that isn’t required when the woman is on continuous monitoring.” (N12)	8
		“…you are observing the baby’s well being and that you are taking care of the woman and not the machine and that the woman is the primary focus. So I think the best outcome is for the woman." (N5)	
		“With EFM you are constantly watching the monitor and you are not as in tune with your patient that’s labouring. They feel more like a patient instead of just a woman going through a process.” (N8)	
	If you’ve experienced a bad outcome while using IA, you are less likely to use it again.	“When you see a bad outcome it can sort of influence you with IA.” (N11)	2
Memory, attention, and decision processes	The decision is an automatic one if there are no risk factors.	“Well it’s automatic if there’s no risk factors that have been identified then it’s automatic [use IA an automatic part of your job or is it something you take time to think about with low risk pregnancies].” (N6)	2
	The decision to use IA is automatic but I’m very conscious of whether or not it’s appropriate.	Well I think about it with the patient in terms of whether or not it’s appropriate for them but it’s automatic to do it if I find that they are low-risk.” (N9)	4
		“No, I automatically do it but it’s not a robotic thing to do like I’m very conscious of whether or not it’s not appropriate. I assume it to be the norm but I am constantly re-evaluating to make sure it is still appropriate.” (N8)	
	The decision to use IA is something I think about with every patient	“Oh I think about it first. I look through all the we have indications for initiating EFM and if she doesn’t belong to one of these indications she can be IA.” (N3)	3
		“With each patient….[take time to think about with low risk pregnancies].” (N10)	
	I can do IA without much thought …it’s a force of habit.	“you know if it’s an urgent situation or fast moving or a slow moving one you are talking and you are doing it at the same time so I’m just reaching for that and holding it on the belly. Like I can do that instantly without belts or wires or anything else. So I think force of habit, believing that’s what we should do and that it is the recommendation.” (N5)	1
	There are not any competing tasks that would influence my decision to use IA or EFM.	“No not really..[any competing tasks or time constraints].” (N8)	3
		“There are things to be done but they can be done as well you know like they can be done in between like we are only auscultating like when we auscultate for a woman in labour it’s only for a minute every 15 so that means you have 14 min every 15 min to do whatever you need to do in between so I think there is opportunity to complete all the long list of nursing things that we do.” (N2)	
	Time is often an issue because if we’re pressed for time we’ll put a mother on Continuous so we can multitask.	“Yes if you’ve got a really busy unit. We’re not any different than any other unit. If it’s really busy it’s unfortunate but sometimes we do use the fetal monitor as a babysitter.” (N6)	8
		“…yes, if somebody’s really busy they might put a monitor on and leave it on because then they can stick their head in and listen.” (N5)	
		“What ends up happening is women are not supported adequately by nurses who are pressured to care for more than one patient at a time and the woman ends up having an epidural and/or augmentation of labour and they end up by default on continuous monitoring.” (N12)	
		“Absolutely the busyness of the floor. If it’s very busy and somehow I end up with 2 labouring patients which I understand are not the guidelines but if it’s really busy I may consider putting a woman on continuous fetal heart monitoring because I can’t get to her.” (N3)	
	Having the proper equipment (Hand held Dopplers & ultrasounds) available encourages the use of IA	“I think having more Doppler’s and one assigned for each room versus the monitor that is there and so prominent right beside the bed.” (N1)	7
		“…we do I think the handheld ones yes. They can cut out with the batteries and that.” (N4)	
		“We could use more Dopplers like we have one Doptone that we can use for in the big tubs that’s safe for using in the water but if we had a few more of those then that would help us too I think. We do have monitors in each of our rooms like we do have that available to us, the big monitors.” (N10)	
	Having easy access to EFM technology directly next to the mothers bed decrease the nurse’s chance of using IA.	“So the monitors are hooked up to a computer and all the computers are connected and the computers are accessible anywhere on our unit…So I think like the central monitoring makes it very easy to like if you are in a room and you are having issues you are not the only one that’s seeing it.” (N2)	5
		“We have portable monitors which do make it easier to do continuous monitoring because they can be up and walking around and in the shower and in the tub and then it also makes it easier because they are waterproof and you can do intermittent and you can go in there and do it that way.” (N8)	
		“…but all assessment areas are set up with monitors and the belts and everything you need and the drawers even have the equipment for the internal monitoring to go on the baby’s scalp so it’s easy to use the monitor but it’s also I mean you use the same equipment to do IA.“ (N5)	
	Missing and broken dopplers discourages the use of IA.	So there’s that and I know this sounds silly but we have handheld Doptones and they are always lost or broken.” (N11)	2
		“…my unit we used to have Dopplers but for some reason all our Dopplers have grown legs and walked off the unit so we use the ultrasound as a Doppler. We don’t hook them up we just hold the monitors to their tummies so it’s the same machine we just don’t put the belts on their tummy.” (N2)	
Social influences	A fellow colleague/clinical leader or physician do/do not influence my decision to use IA if they detect something I’ve missed	“I find that other nurses think if I’m taking over from another nurse like at change of shift if that nurse has had the patient on the monitor, but there has not been any medical indication for continuous monitoring it’s difficult for me to take her off in terms of the patient saying ‘well how come I needed to be on before?’ without seeming like I’m undermining my colleague’s judgment.” (N11)	9
		“I would say it would be if your team members your nurse colleagues and the obstetrician that’s monitoring the progression of labour.” (N12)	
		“Unless it was specifically ordered by a physician no.” (N8)	
	I do (don’t) discuss my cases with other colleagues	“No…[don’t discuss a case with your colleagues].” (N3)	12
		“Yes…[discuss a case with your colleagues before deciding whether to use IA].” (N4, N8)	
		“Yes we do. We look at our sheets that we get from our physicians and like often if we know they are coming in or maybe they’ll just show up we’ll pull it and between the 2 nurses there we’ll say what do you think and again you know we really there’s not all of us that are using it either right because this is a new thing right.” (N7)	
		“No for me I would be able to make that decision on my own but I guess if I had questions about it I could easily go to another person.” (N10)	
	The labouring woman’s emotions do/don’t greatly influence my decision to use IA	“For me it’s just the data, again just talking to the mom, looking at her comfort level.” (N7)	12
		“I don’t think they [woman’s emotions] play into that at all.” (N1)	
		“Like if she was really concerned or if she was frightened or if her last baby had something happen and she felt more comfortable having the monitor on then that would be something that we would certainly try to address for her.” (N10)	
		“Oh yes they do. If the patient is exhausted and tired and doesn’t want to walk around because I do encourage ambulation and some patients think I’m cuckoo like I’m tired I don’t want to walk around….Because there are women who have said to me I want to try and sleep and I says yes…if they don’t want me to bother them I will put the EFM on. It’s not my first choice but if they want to sleep and they don’t want to be bothered and me looking for the fetal heart every 15 min I’ll say you know you are considered in active labour can I throw the monitors on you? With their permission and the majority of time they say yes.” (N3)	
	Having the support of the hospital policies and physicians greatly influences my use of IA	“The obstetricians here are really supportive of us using IA, most of them are very current and very supportive. Some of the family doctors that I’ve worked with in the past would want you to have them on the monitor more often and I’ve had some of my obstetricians that don’t want a monitor on and if you could manage the labour without a monitor that’s even better. So they encourage us to use IA. I guess it depends on who you are working with on that case too.” (N10)	8
		“Yes. I think this is a big influencing factor especially to young, new grads that have a new nursing degree and this is their first job you know versus someone like me who has only worked there for a shorter period of time, I’ve got years of experience with it, it’s that if your team leader or your mentor the person that’s orientating you they for the most part we teach our young kind of what we know, what we do, and so you just pass it on so you just pass it on so definitely like if the team leader says ‘oh I know she’s still in that normal category but I’m not so sure put that monitor on’ you are going to cause a lot of personal, political, professional strife if you start contradicting what a leader has asked you to do." (N5)	
		“That is not the model if you like that is encouraged by nursing management or by obstetricians for different reasons. There’s a whole politics and a pressure put on individual primary care nurses to not be continuously in the room with one woman because if you are then you cannot look after more than one patient safely and so IA requires one-on-one nursing and the skill to support the woman in the room.“ (N12)	
	It’s difficult when physicians question/s-guess your decision.	‘I mean I’ve been there you know done that wore the T-shirt with continuous fetal monitoring where everything is fine and then you are pushing and then boom. We know it happens and can we predict it more with continuous I don’t think you can if everything has been fine but I think it’s that piece that really bothered me was the clinician saying to the nurse ‘why wasn’t she on the monitor?’ and the nurse just said because she’s low-risk—IA!" (N11)	3
		"The biggest gap is that SOGC comes up with guidelines that are really evidence-based and clinicians go ‘yea well I’m not doing that’. Okay well it is the national organization of your colleagues or of our colleagues."(N5)	
		"Obstetricians can become quite concerned because they have to rely on your assessment not only of the fetal heart rate but the contraction pattern and most labours are actively managed. There is a pressure to have women in and out and delivered within a kind of unspoken time limit." (N12)	
	The cultural pressure to be part of the team at the nurses’ station, using IA can be isolating	“There is a culture if you like also. I’m very interested in the anthropology of you know the way nurses censor each other and if you are not sitting at the desk watching monitor screens and you are in the room with the woman you can be censored in many ways non-verbally even because you’re not involved in sitting having discussions at the nursing station rather than in the room with the woman which is a little depressing at times. You are not seen as part of the team if you are in a room all the time quote unquote.” (N12)	2
Behavioural regulation	A way to encourage IA use is review of the correct procedures to re-familiarise all staff (nurses, family physicians and obstetricians)	"Like every so often we do have, what we call, a cycle review on the procedures that are more commonly used. I think if say the policy on that is that is one of those things that were reviewed on a yearly basis that would encourage people to do it and it really makes sense." (N2)	7
		"Well it’s definitely the education factor because what I’m finding now is now that we are into the more OB program and we’ve got the fetal health surveillance learning package and that the more I educate staff the more they understand. It’s getting them away from the electronic." (N4)	
		"Just continued education for staff around the benefits sometimes I’m not convinced that people see there’s a benefit. I think still some of my staff see continuous fetal monitoring as being more preventative and IA as being more reactive. I don’t know it’s a hard sell to be honest." (N11)	
	Better communication so that the team is more supportive of the nurses assessment would improve the use of IA	“I think there would have to be really very frank discussion interdisciplinary discussions and a willingness for the obstetricians who, of course, along with the hospital take on the liability risk. So the hospital and the obstetricians would have to be willing to come onboard with that and to not have this false sense of security in my view that you know a continuous readout is better and safer and more reliable than a well-trained observant nurse.” (N12)	3
		“I think it’s their perception often needs to be corrected about options that are available. So for example if a resident comes in and says ‘okay is she on the monitor?’ I’ll say ‘no, no we don’t’ need to use the monitor we’re just using IA and the fetal tone has been great and I’ve heard accelerations and it’s been fine’. So I use it as an educational opportunity.” (N11)	
	More modelling techniques would help	"The policy is good I just think as in the previous question it’s around development of teaching tools and modelling and encouraging others to develop the confidence in it." (N9)	6
		"…to make the steps necessary for that is I believe lead by example because it’s very much a culture thing in that a lot of nurses like the EFM, they strap it on and they don’t have to worry about it and they let it go so it’s very much a trend and it’s something that needs to catch on either by leading by example or having more push for IA would help in our facility." (N8)	
	Better communication between nurses and physicians so that Physicians are more supportive of the nurses assessment would improve the use of IA	"I think we have to change that it’s a paradigm shift right. I think we’ve got to change the mentality of people from putting them on EFM automatically and I do believe that in order to do IA successfully and get these patients walking around more we need more Doppler’s. We don’t have any." (N3)	3
		"I think there would have to be a really very frank discussion, interdisciplinary discussions, and a willingness for the obstetricians who, of course, along with the hospital take on the liability risk. So the hospital and the obstetricians would have to be willing to come onboard with that and to not have this false sense of security in my view that you know a continuous readout is better and safer and more reliable than a well-trained observant nurse." (N12)	

Nurses reported that IA was part of their fetal surveillance plan and that because they had been performing IA for so long, it was just what they did (domain: *Nature of behaviour*). However it was mentioned that other nurses may be ‘set in their ways’ and will always do what they’ve done whether it was IA or EFM. Nurses did note that while they would use IA in situations where the mother was healthy and there were no complications, there were circumstances (e.g. transition to second stage labour, epidural, use of Oxytocin) whereby IA was just not appropriate (domains: *Nature of behaviour; Behavioural regulation*).

Most participants stated that they were very comfortable using IA with a healthy woman in labour and reported being confident in their ability, but they also noted that it was very difficult to use IA if the mother was already being monitored using continuous EFM (*Beliefs about capabilities)*. The nurses also varied in their responses about the ease of deciding to use IA. For example, nurses reported that while using IA is their default decision, they were very aware that it might not be appropriate for every mother they see (domain: *Memory, attention and decision processes, Nature of behaviour)*. Others noted that the decision to use IA is the default decision as long as there are no risk factors. However some nurses reported that the decision to use IA is something that they consciously think about with every patient (domain: *Memory, attention and decision processes)*.

The ease or difficulty of using IA was also attributed to accessibility of equipment, time management concerns and the requirement of multitasking (domains: *Belief about capabilities, Environmental context and resources)*. The nurses reported that having easy access to handheld Dopplers and ultrasounds encourages the use of IA while having the EFM technology directly next to the mother’s bed and/or missing and broken Dopplers decreases the nurses’ opportunity to use IA. They stated that using IA takes more time than other forms of fetal surveillance *(Beliefs about consequences)* and they are reluctant to use it when they have to monitor more than one mother and multitask (*Environmental context and resources).*

When identifying the possible consequences of using IA as the predominant method of fetal surveillance the nurses’ responses varied considerably. While some nurses reported that using IA reduces unnecessary interventions and gives the nurses opportunities to support the mother, others identified potential legal concerns due to the lack of a printed EFM monitor strip should something go wrong. They also noted that if they had experienced a bad outcome in the past with IA they would be less likely to use it in the future. However, most did report that IA allows the nurses to be more focussed on the mother and baby, compared with continuous monitoring (*Beliefs about consequences)*.

Other factors that influenced nurses’ use of IA focussed on the domain *Social influences.* Nurses mentioned that the mother in labour and family greatly influence their use of IA such that if the mother was concerned or frightened and preferred the continuous EFM then they would use it instead of IA. If the mother was tired, wanted to sleep, and did not want to be bothered every 15 min with IA surveillance, then the nurse would reluctantly use continuous EFM. Others reported that they would address the parental concerns, such as changes in heartbeat rate, and continue with IA. The nurses reported that parental concerns could be used as an opportunity to inform the mother of what is actually happening during labour. Nurses also reported that colleagues might influence their decision to use IA. Some nurses reported that they do not discuss cases nor do other health professionals influence them, whereas others reported that they often discuss case with fellow nurses, specifically if something went wrong during the monitoring and they wanted to debrief with fellow colleagues (*Social influences*). It was also mentioned that there is a cultural pressure to be part of the team at the nurses’ station that limits use of IA, because using IA isolates the nurse away from their colleagues. Nurses also reported difficulty with using IA if an obstetrician questions the nurses’ initial assessment (*Social influences*) and that having the support of hospital policies and physicians would encourage the use of IA (*Social influences, Behavioural regulation*).

When asked about ways to increase the use of IA most of the nurses mentioned better communication between the nurses and physicians so that the physicians are more supportive of the nurses’ assessment (*Behavioural regulation, Social influences*). Additionally, they mentioned review of standard policies and procedure with the entire team so that everyone is in agreement with the use of IA as well as modelling to ‘lead by example’ when encouraging the use of IA as the predominant method of fetal surveillance (*Behavioural regulation, Beliefs about capabilities, Social influences)*.

### Domains less likely to inform intervention design to change behaviour

Five domains were identified as less like to inform intervention design to improve birthing unit nurses’ use of IA as a predominant method of fetal surveillance: *Knowledge, Skills, Social/Professional Role and Identity, Motivation and goals, Emotion* [Table 3]. The nurses were aware of the SOGC guidelines and all believed the guidelines were strongly based in the evidence (*Knowledge*). They identified that skills required to use IA are not different than any other techniques to monitor a baby (*Skills*). Most of the nurses interviewed reported that using IA was part of their role as a BU nurse (*Social/professional role and identity*) and reported that the training and practice they receive as a BU nurse influences their use of IA. Further, the nurses reported that using IA was important to them (*Motivation*), and they were not worried when using IA in the appropriate circumstances (*Emotion*).

**Table 3:** Summary of belief statements and sample quotes from Birthing Unit Nurses assigned to the theoretical domains identified as not relevant to changing fetal surveillance behaviour

Domains	Specific belief	Sample quote	Frequency out of 12
*Knowledge*	I am aware of guidelines. (Provincial/National)	“…the SOGC guidelines about fetal surveillance which encourages us to be using auscultation with low-risk pregnancies…” (N1)	11
		“We follow the SOGC guidelines.” (N4)	
		“Well I know the SOGC has Guidelines.” (N5)	
		“Our hospital tries to follow More OB Guidelines.” (N8)	
	There is evidence to support the use of IA guidelines.	“I believe they are evidence-based and I think the evidence is good in that like I’ve seen the trend and that it tends to be focusing on the fact that increased continuous fetal monitoring generally it doesn’t improve fetal well being. It simple increases intervention.” (N5)	12
		“Yes [they’re evidence based]. I have read the background information so I understand that there has been a lot of Cochrane reviews and things like that so yes I do trust it.” (N11)	
		“Oh I do believe they are evidence-based. They have had many studies and proven similar outcomes between fetal monitoring and intermittent auscultation.” (N3)	
		“Yes I think that they are and I think that the evidence is that outcomes are not necessarily improved by continuing fetal monitoring. I think that the risk of C-section related to increased interventions is an outcome of EFM.” (N6)	
*Skills*	There are skills requires to use IA but they are no different than any other technique to monitor a baby.	“Yes there certainly is a skill set to that [IA] but that’s with any sort of fetal monitoring including continuous monitoring…” (N10)	12
		“I don’t think so. I think you need to know how to interpret things. I don’t think the skill is different it’s the interpretation of what you are listening to…same expertise that you would need for any kind of listening to the baby.” (N1)	
		“I think it’s just the general experience of understanding the labour process and what to expect in terms of fetal heart rates and patterns during the labour process." (N11)	
Professional/Social Role & Identity	Using IA is part of my job as a Birthing unit nurse.	“I think I’m doing more of a job that way because I am more aware of her and I am more present and I am in the room as opposed to being at a desk watching a strip.” (N8)	11
		“Absolutely…[I’m doing my job by using only IA].” (N9)	
		“I think I am [doing my job]. I think I’m being a little bit extra when I’m using IA because it demands that you are more present with the patient if you do it versus if you just hook them up to the monitor and leave them on the monitor continuously. So I feel like I’m giving a little bit extra effort when I do it.” (N2)	
	The training and practice we receive Birthing Unit nurses influences my decision to use IA	“My preceptor training was very, very good and she paid a lot of attention to detail and she really made it clear to me that it’s an option and when it’s a safe option so I think it’s just who I was trained by when we initially got hired on the unit that makes the decision so easy for me to make because she made me feel like this is something that we are allowed to do, it doesn’t happen very often, but we are allowed to do it so I think it’s my training.” (N2)	6
		“I think being trained in the More OB is beneficial and I think personally a lot of reading and extracurricular done on my own has been more directed toward the midwifery model of care and the hands off approach and I think that is what makes me push for and make sure I’m more aware of the importance of doing IA.” (N8)	
	As a nurse we have a standard of practice that guides our care of a labouring woman.	“…our moral bead that we’re involved in…[is special to our training as a Nurse that influences use IA].” (N1)	5
		“…that it is the preferred method and it is within our standards of practice…” (N11)	
		“I just think if you have like I think as a nurse you all know what a low-risk pregnant woman presents as and when to use IA.” (N2)	
*Motivation and goals*	Using IA is important to me.	“Very important. You’ve got better patient satisfaction. You’ve got equal outcomes. The babies do just as well with IA as they do with fetal monitoring; everybody is happier.” (N6)	9
		“I actually think it’s very important. I think we’ve got to get nurses comfortable in doing it though. I think because the climate has been to put them on EFM and I believe we really need to get away from that.” (N3)	
		“Yes [it’s important] I think because I believe in it.” (N5)	
	IA is a top priority for me as long as it done safely	“I think it’s very important to use it because especially if it’s a woman who is trying to go ‘au naturale’. ” (N2)	3
		“For un-medicated births I would say it’s very much the top priority to be able to do IA in the way that you know it is safe.” (N12)	
*Emotion*	I’m not worried when using IA.	“No, [I’m not worried]. If it’s low-risk and there’s nothing and healthy there should be no reason why we have to change our plan of action.” (N7)	12
		“Whether I’m on the monitor or not, so [there’s] no difference for me so using IA does not make me worry more." (N11)	
		“No [using IA with a healthy woman having a low risk pregnancy ever evoke worry or concern in me]. ” (N9)	

Note: “N#” indicates sample quote by Nurse

## Discussion

This study applied the TDF (v 1.0) [30] to help understand the influences on IA use by BU nurses with low risk women in labour. The results show that the reported influences on the nurses’ use of IA and thus of a specific fetal monitoring practice could be coded into seven of the 12 TDF domains: *Beliefs about capabilities, Beliefs about consequences, Environmental context and resources, Memory, attention and decision processes, Social influences, Behavioural regulation,* and *Nature of the behaviour*. The Birthing Unit nurses’ views about fetal surveillance reported within the seven domains centred on two main issues*.*

Firstly, while nurses identified that they were very comfortable and confident in their ability to use IA (*Beliefs about capabilities*), they found it difficult to follow through with actually using it because of the influence of expecting mothers, fellow nurses, obstetricians or hospital administration (Beliefs about capabilities, Social influences). A number of studies have reported that nurses stated the biggest advocate for their use of IA was a team lead who strongly supported the use of IA [2, 9, 39]. Our study supports these findings and further expands it to obstetricians and hospital management (*Beliefs about capabilities, social influences, Behavioural regulation*). Nurses may be the health professionals who make the decision to use or not use IA but hospital policies and obstetricians heavily influenced the decision (*Behavioural regulation; Social influences*). In addition, whilst the Society of Obstetrics and Gynaecology of Canada guidelines state surveillance should occur every 15–30 min first stage of labour [4], in practice most hospitals aim for 15 min interval because 30 min is believed to be a long time when active labour is progressing. Consistency with hospital policy and national guidelines would likely better facilitate the use of IA. If they do not have the support of hospital management and the physicians they work with, nurses identified that it was difficult to use IA (*Social Influences, Beliefs about capabilities*).

In 2002, the Society of Obstetrics and Gynaecology of Canada launched an obstetric patient safety program, ‘Managing Obstetric Risk Efficiently’ (MORE^OB^), where the end-point was to change the culture to promote patient safety [40]. MORE^OB^ developed a model of care that promoted inter-professional teamwork to ensure trust and respect for all team members [40]. By 2012, the program had been implemented in 10 provinces and territories and in 74 hospitals in Ontario and reported marked success in lowering litigation costs and improving patient safety culture of obstetrical units [41]. However, our study shows that lack of team support remains a concern for the nurses with respect to using IA. It may be of benefit to examine the components of MORE^OB^ that improved obstetrical culture about patient safety and investigate whether they could address the culture changes about using IA as the primary method of fetal surveillance [42]. Critical to ensuring the proper use of IA is to have everyone involved in the mother’s care supportive of the use of IA. This could reduce negative aspects for nurses making the initial decision to use IA (time consuming, legal concerns associated with IA; *Beliefs about consequences).*

The second main issue was that nurses noted that accessibility to equipment could act as both a barrier and enabler to the use of IA (*Beliefs about capabilities, Environmental context and resources*). Currently, electronic fetal monitors are often located in the labour rooms, making continuous monitoring easier, and some are connected to a central monitoring display. Missing and broken Dopplers prevent the nurse from using IA. However, according to guidelines, one can use a manual fetoscope to perform IA (i.e., the Doppler is not a requirement for IA), suggesting that an alternative form of IA is possible. It is unclear whether improved access to the perceived necessary equipment would lead to increased use of appropriate IA. However, to address this perceived barrier, accessibility to hand-held Dopplers would help.

These two issues reported by nurses reflect the way participants articulated their experiences with fetal surveillance and may have implications for intervention delivery. By addressing the key issues in an intervention that are based on the TDF and specific belief statements, the intervention could have a greater coherency for participants and, as a result, encourage engagement.

### Limitations

While this study has provided valuable insight into the factors that may influence fetal monitoring practices, there were several limitations. First, similar to other studies that use the TDF [16, 24, 25, 28, 34, 43, 44], identification of themes represent clinicians’ views about what might influence their fetal surveillance practice. Although interview studies are required in the exploratory stages of research in this field, other research designs would be required to establish which of these factors are actually key to changing practice. Because of the nature of the TDF, the scope of the data collection and analysis were limited to the behaviour under investigation and the potential barriers and enablers to enacting that behaviour, rather than general view about fetal surveillance or other topics that may present themselves in the interview. Alternate forms of qualitative analysis (grounded theory, thematic analysis) may prove useful in capturing that information. However, as previously mentioned, this was not the scope of this study.

Secondly, participant recruitment began with individuals replying to a listserv mail out. It is likely that we received responses from individuals who felt strongly about fetal surveillance practices as is evident from the common themes and limited contradictory statements from the participants. We do not know if non-responders have different views about IA or other fetal surveillance practices and cannot necessarily generalise our findings to all birthing unit nurses. However, this study will be used to guide a larger questionnaire study to identify psychological determinants of the nurses’ fetal surveillance practice. This will provide us with the opportunity to confirm or rebut the findings from the interviews and address the previously mentioned two limitations.

While this study under review to the journal, an update of the Cochrane Systematic review was published, reporting that some of the evidence around continuous EFM and IA had changed [45]. In particular, continuous monitoring was associated with fewer fetal seizures and no difference in cases of cerebral palsy but both are rare events. However, continuous EFM was still associated with increased caesarean sections and instrumental births [45]. Continuous EFM can also restrict the woman’s movement, makes changing positions difficult during labour and the birthing pool cannot be used [45]. Since continuous EFM may negatively impact the woman’s coping strategies, choice of fetal surveillance may be more dependent on the woman’s individual needs and wishes about monitoring the baby’s wellbeing rather than clinical outcomes.

Finally, while we interviewed the professional group believed to be responsible for decision-making, our study identified others who may influence their decision (patients, clinical leads, obstetricians, managers; *Social Influences*). It would have been ideal to include these groups in the interviews to explore different perspectives on the issue. However, our study was directed at the nurses’ perspectives since they are directly responsible for the decision at the initial point of contact with the patient and perform the behaviour under investigation.

## Conclusion

This study examined birthing unit nurses’ fetal surveillance practices in a systematic way, drawing on a theoretical framework of behaviour change to inform possible components of interventions to improve fetal monitoring practice. It is one of the first studies to use the TDF with the nursing profession. Our results identified potential influences upon fetal monitoring behaviour of birthing unit nurses. Our findings are being used to develop questionnaire materials for a predictive study to further explore determinants of fetal surveillance practices. In addition, the results be used to develop an intervention using mapping directly from the domains [46] to behaviour change techniques [36]. By using the TDF, our study provides a theory-driven basis to identify likely influences on nurses’ behaviour to encourage Intermittent Auscultation where appropriate for healthy, low-risk, women in labour.

## Additional file

Additional file 1: Patey et al. fetal surveillance interview guide contains that Semi-structured interview guide, based on the TDF, used in this study. (PDF 149 kb)

## Abbreviations

BU: Birthing Unit
EFM: Electronic Fetal Monitoring
IA: Intermittent auscultation
N#: Nurses
TDF: Theoretical domains framework
