# Supplementary material for: Intermittent auscultation versus continuous fetal monitoring: exploring factors that influence birthing unit nurses’ fetal surveillance practice using theoretical domains framework
Source: BMC Pregnancy Childbirth. 2017 Sep 25;17:320. doi: 10.1186/s12884-017-1517-z (PMC5613395; doi:10.1186/s12884-017-1517-z)
Supplement: Additional file 1: — Patey et al. fetal surveillance interview guide contains that Semi-structured interview guide, based on the TDF, used in this study. (PDF 149 kb) [file 12884_2017_1517_MOESM1_ESM.pdf]

Semi-Structured Interview Schedule for Fetal Monitoring in Healthy Low Risk Women During Labour

Introduction: *The general aim of the interview is to help us understand how you make decisions about fetal monitoring for a healthy woman with a low risk pregnancy. We want to know what influences your decision to use Intermittent Auscultation instead of Electronic Fetal Monitoring. There are no right or wrong answers; we are trying to understand how different clinicians approach this issue, so please answer frankly.*

I'd like to start with some basic questions about your practice:

1. What type of setting do you practice in? (community vs. academic)
2. How many years have you worked in the Birthing Unit?
3. Please describe the typical process for a healthy woman coming in to deliver a baby?
4. What is your definition of a Low risk pregnancy?
5. On average, how many women do you see per week would you define as low risk? Percentage

Thank you.

For the rest of the interview, I have some slightly more specific questions about what influences your use of IA for healthy women with low risk pregnancies. Some questions may seem repetitive, but please bear with me as the questions are derived from different models of human behaviour and we are trying to figure out which models best apply in this area. Keep in mind, then when I ask about IA, I am referring to it being the primary method of fetal surveillance during the labour

Are you ready to get started?

Set the scene re: I'm asking about the situations where IA is the predominant method of fetal surveillance during labour in healthy women with "**low risk pregnancy**" – I'd like you to think about that for a moment…

Knowledge

6. Are you aware of any guidelines (national, provincial or institutional) about fetal monitoring in low risk pregnancies?
7. What is your understanding of the guidelines? **(Prompt –what do they say?)**
8. Do you believe these guidelines to be evidence based? What is your interpretation of the evidence?

Skills

9. How much expertise or experience do you think one needs to have to use IA as a method of fetal surveillance in labour?
10. Are there any skills that are necessary to be considered competent in the use of IA? What are they?

Nature of the Behaviour

11. Is IA a part of your fetal surveillance for low risk women? In what clinical circumstances?
12. Are you comfortable using IA in this patient population? WHY or Why not?

Memory, Attention and Decision Processes

13. What thought processes guide your decision to use IA for fetal surveillance in this low risk population? **(Prompt: "What goes through your mind?")**
14. Is making a decision to use IA an automatic part of your job or is it something you take time to think about with low risk pregnancies? **(Prompt routine, automatic)**
15. Is it typically an easy or difficult decision to make? **(Prompt: Weigh pros and cons etc.)**

Social/Professional role & identity

16. If you are monitoring a healthy labouring woman and you only use IA, do you think you're doing your job?
17. Is there something that is special to your training as a Labour and Delivery Nurse that influences your decision to use IA with a healthy woman having a low risk pregnancy? **(Prompt: professional training, a protocol, an order set, other technologies)**

Environmental Context & Resources

18. What aspects of the Birthing Unit environment (physical) influence your choice of fetal monitoring of a healthy woman having a low risk pregnancy?
19. Are there any resource factors in your Birthing Unit that influence the choice of fetal monitoring you use?
20. Are there any competing tasks or time constraints that might influence whether or not you use IA with a healthy woman having a low risk pregnancy?

Motivation and Goals

21. Considering your other priorities in this clinical situation, how important do you think it is to use IA as the predominant method of fetal surveillance with a healthy woman having a low risk pregnancy?
22. Are there any personal incentives for you to use IA in healthy women having a low risk pregnancy? **(Prompt: goals within yourself? external?)**

Beliefs about Capabilities

23. How easy or difficult is it for you personally to use IA as the predominant method of fetal surveillance with a healthy woman having a low risk pregnancy?
24. If difficult, what issues have they encountered that make fetal monitoring using IA problematic?
25. If easy, what has help to make the use of IA easier?
26. Are you confident that you are able to properly assess the fetus using IA?

Social Influences

27. Would any other team members influence whether or not you use IA as the predominant method of fetal surveillance with a healthy woman having a low risk pregnancy?

(<u>Prompt</u>: who else; Other clinicians; medical staff including fellow nurses and residents/fellows; relatives)

28. Do you ever discuss a case with your colleagues before deciding whether to use IA as the predominant method of fetal surveillance with a healthy woman having a low risk pregnancy?
29. Do your colleagues generally agree with you on this issue?

Emotion

30. How do the labouring woman's emotions affect your decision to use IA as your primary fetal surveillance method? **Prompt: "If a woman is upset or distressed would that influence what method of surveillance you use?"**
31. Would using IA with a healthy woman having a low risk pregnancy ever evoke worry or concern in you?

Beliefs About Consequences

32. What do you think will happen if you used IA as your predominant fetal surveillance method during labour, both positive and negative? ***(prompt: to patients, to colleagues, yourself, short and long term)***
33. What are the drawbacks to using IA as the predominant method of fetal surveillance on a low risk woman in labour?
34. What are the benefits to using IA as the predominant method of fetal surveillance on a low risk woman in labour?

Behavioural Regulation

35. In an ideal world, where anything is possible, what would see as best process of fetal monitoring in a healthy woman with a low risk pregnancy?
36. What are some ways/steps of working that would encourage the use of IA as the primary method of fetal monitoring in healthy women during a Low-risk pregnancy?
37. If you wanted to implement changes in your own practice (individual/team setting/practice setting) to encourage the use of IA for a healthy woman with a low risk pregnancy, what do you think would be the steps necessary to do this?

That's all the questions I have for you; has anything occurred to you about this topic that we haven't asked about?

Thank you!
